# Metformin-Clinical Pharmacology in PCOs

**Published:** 2015

**Authors:** R Dumitrescu, C Mehedintu, I Briceag, VL Purcărea, D Hudita

**Affiliations:** * “Carol Davila” University of Medicine and Pharmacy, Bucharest, Romania; ** Gynecology Department, “Nicolae Malaxa Hospital”, Bucharest, Romania; *** Gynecology Department, “Dr. I. Cantacuzino Hospital”, Bucharest, Romania

**Keywords:** PCOs, infertility, metformin, diabetes mellitus, insulin resistance

## Abstract

Oligo-anovulation, hyperandrogenism and insulin resistance characterizes polycystic ovary syndrome (PCOs). Metformin is the oldest insulin sensitizer used in the management of type 2 diabetes mellitus. In PCOs, metformin decreases the serum lipids, androgen and insulin; induces ovulation and regular menstrual cycle; increases the pregnancy rate.

**Metformin** is a first-line therapy for type 2 diabetes mellitus (T2DM, formerly “non-insulin-dependent diabetes mellitus”), and is one of the most commonly prescribed drugs worldwide, indicated in the guidelines issued by the American Diabetes Association and European Association for the Study of Diabetes. As a biguanide agent, metformin lowers both basal and postprandial plasma glucose (PPG) [**1**,**2**]. Metformin works by inhibiting the production of hepatic glucose, reducing intestinal glucose absorption and improving glucose uptake and utilization. Besides lowering the blood glucose level, metformin may have additional health benefits, including weight reduction, lowering plasma lipid levels, and prevention of some vascular complications [**3**]. Metformin is also used for other indications such as polycystic ovary syndrome (PCOs) [**1**].

Metformin hydrochloride is a white to off-white crystalline compound with a molecular formula of C4H11N5 • HCl and a molecular weight of 165.63. Metformin hydrochloride is freely soluble in water and is practically insoluble in acetone, ether, and chloroform. 

Metformin hydrochloride tablets for oral administration contain 500 mg, 850 mg or 1000 mg of metformin hydrochloride. 

**Mechanism of Action**

Metformin is an antihyperglycemic agent that improves glucose tolerance in patients with type 2 diabetes, lowering both basal and postprandial plasma glucose. Its pharmacologic mechanisms of action are different from other classes of oral antihyperglycemic agents. Metformin decreases hepatic glucose production, decreases intestinal absorption of glucose, and improves insulin sensitivity by increasing peripheral glucose uptake and utilization. Metformin does not produce hypoglycemia in either patients with type 2 diabetes or normal subjects and does not cause hyperinsulinemia. With metformin therapy, insulin secretion remains unchanged while fasting insulin levels and daylong plasma insulin response may actually decrease.

**Pharmacokinetics**

**Absorption and Bioavailability**

Given under fasting conditions the absolute bioavailability of a metformin hydrochloride 500 mg tablet is approximately 50% to 60%. Studies using single oral doses of metformin hydrochloride tablets of 500 mg to 1500 mg, and 850 mg to 2550 mg, indicate that there is a lack of dose proportionality with increasing doses, which is due to decreased absorption rather than an alteration in elimination. Food decreases the extent of and slightly delays the absorption of metformin.

**Distribution**

Metformin is negligibly bound to plasma proteins in contrast to sulfonylureas, which are more than 90% protein bound. Metformin partitions into erythrocytes, most likely as a function of time. At usual clinical doses and dosing schedules of metformin hydrochloride tablets, steady-state plasma concentrations of metformin are reached within 24 to 48 hours and are generally <1 mcg/mL. During controlled clinical trials of metformin, maximum metformin plasma levels do not exceed 5 mcg/mL, even at maximum doses.

**Metabolism and Elimination**

Intravenous single-dose studies in normal subjects demonstrate that metformin is excreted unchanged in the urine and does not undergo hepatic metabolism (no metabolites have been identified in humans) or biliary excretion. Renal clearance is approximately 3.5 times greater than creatinine clearance, which indicates that tubular secretion is the major route of metformin elimination. Following the oral administration, approximately 90% of the absorbed drug is eliminated via the renal route within the first 24 hours, with a plasma elimination half-life of approximately 6.2 hours. In blood, the elimination half-life is of approximately 17.6 hours, suggesting that the erythrocyte mass may be a compartment of distribution.

Metformin is not metabolized [**4**] and is excreted unchanged in the urine, with a half-life of ~5 h [**5**]. The drug is widely distributed into the body tissues including the intestine, liver, and kidney by organic cation transporters [**5**]. There is a large interindividual variability in metformin pharmacokinetics as measured by differences in trough steady-state metformin plasma concentration ranging from 54 to 4133 ng/ml [**6**].

Insulin resistance appears to be the fundamental common pathway to disease amongst women with PCOs. Women with PCOs have normal insulin molecules and the insulin receptor on cells appears to be normal. However, it appears to be a post-receptor deficit, in relation to the downstream cellular effects of what happens after insulin binds to the insulin receptor, meaning that the molecular cascade of intracellular events has a level of impairment, leading to a post-receptor “intracellular” resistance to insulin. Since there is a relative insulin resistance, women with PCOs produce higher levels of insulin than they otherwise would have. These increased circulating levels of insulin have direct effects on the ovaries, and the increased insulin levels also release other factors—notably insulin-like growth factor 1 (IGF-1) from the liver—which, in turn, exerts an effect on the ovary. The impact of higher levels of insulin and IGF-1 on the ovary is for the ovary to release higher levels of testosterone. All these hormones—including insulin, IGF-1 and testosterone—prevent the growth of ovarian follicles through to ovulation, leading to an accumulation of small ovarian follicles less than 10 mm diameter that do not progress through to ovulation.

Rotterdam criteria for the diagnosis of PCOs :

• * At least twelve small follicles 2-9 mm in at least one ovary;

• * Symptoms or biochemical evidence of hyperandrogenism;

• * Anovulation or oligo-ovulation with fewer than nine menstrual periods at every 12 months.

Metformin is a very suitable alternative to clomiphene as a first line ovulation induction treatment for non-obese women with anovulatory PCOs. In fact, metformin carries some potential advantages over clomiphene, including no known adverse endometrial effect whilst endometrial thinning could reduce embryo receptivity for some women using CC [**7**,**8**], no known increase in multiple pregnancy rate (unlike that associated with CC) and thus no requirement for the inconvenient and costly monitoring of ovulation induction cycles (that many fertility clinics insist upon for CC), and no concern regarding the long term adverse effects on the ovaries, contrasting with the lingering concern over an increased risk of ovarian cancer seen in some cohort studies of women using CC, particularly serous ovarian cancer [**9**] and amongst those using long treatment courses [**10**].

There is a growing evidence base that metformin response is better in women with PCOs who have a lower BMI [**11**,**12**]. Clinical pregnancy rate was significantly higher in women receiving dual therapy versus clomiphene alone.

Metformin may accumulate in certain tissues at higher concentrations than in plasma [**13**]. The passive diffusion of metformin into cells is limited [**14**], the main transport is the organic cation transporter 1-3 or multidrug and toxic compound extrusion type transporters which are able to internalize metformin as described in gut, hepatocytes, renal tubular epithelial cells and reproductive tissues [**15**]. One of the direct effects of metformin identified is to inhibit the activity of the respiratory electron transport chain in mitochondria [**16**] and to activate the cytoplasmic protein kinase known as AMP-activated protein kinase (AMPK) [**17**]. AMPK is an important sensor of cellular energy homeostasis and is sensitive to the AMP: ATP ratio [**18**,**19**]. It can be noted that several studies have also demonstrated that metformin might act independently of AMPK [**20**-**22**]. A deficiency in ATP activates AMPK leading to increased energy production including glucose and lipid catabolism and an inhibition of energy consuming processes such as protein, fatty acid and cholesterol synthesis.

**Metformin and central control of reproduction**

Fertility is centrally controlled at least in part by gonadotropin-releasing hormone (GnRH) neurons that are located in the hypothalamus. These neurons secrete GnRH, which stimulate the luteinizing hormone (LH) and follicle stimulating hormone (FSH) secretions by the pituitary. GnRH neurons are responsive to numerous stimuli including peripheral metabolic signals such as glucose and leptin [**23**,**24**]. AMPK has been widely studied in the brain as it is involved in regulating food intake, a function regulated by the hypothalamus [**25**-**28**]. The various anorectic signals (leptin, insulin, glucose) reduce AMPK activity whereas the orexigenic signals (ghrelin, neuropeptide Y) increase AMPK activity which permits the regulation of food intake [**29**,**30**]. Metformin inhibits AMPK activity in the hypothalamus and neuropeptide Y neurons in the brain, which explains the appetite suppressing nature of AMPK [**31**]. In the same region of the brain, the functioning of neurons to kisspeptin and to GnRH also seems to be AMPK dependent. Therefore, AMPK is a key participant in the operation of important reproductive neuron regulators.

It works mainly by suppressing excessive hepatic glucose production, through a reduction in gluconeogenesis [**32**]. The other potential effects of metformin include an increase in glucose uptake, an increase in insulin signaling, a decrease in fatty acid and triglyceride synthesis and an increase in fatty acid β-oxidation.

Metformin may also increase glucose utilization in peripheral tissues, and possibly reduce food intake and intestinal glucose absorption. As metformin does not stimulate endogenous insulin secretion, it does not cause hypoglycemia or hyperinsulinemia, which are common side effects associated with other antidiabetic drugs.

Metformin has insulin-lowering effects by improving insulin sensitivity and, in turn, can decrease circulating androgen levels. In addition, it also plays a critical role in the treatment of PCOs, because women with PCOs are at an increased risk of insulin resistance [**33**]. Indeed, metformin improves insulin-mediated glucose disposal in women with PCOs [**34**]. Thus, metformin has become one of the key drugs in the treatment of PCOs.

The molecular mechanisms underlying the metformin action appear to be complex and remain a topic of considerable debate. However, there is a general agreement that the administration of metformin results in the phosphorylation and activation of AMP-activated protein kinase (AMPK) in the liver, which in turn may lead to diverse pharmacologic effects, including inhibition of glucose and lipid synthesis [**2**,**35**].

Metformin alone decreases fasting plasma glucose concentration to 4–4.5 mM and HbA1c by 1.5–2.0% in patients with type 2 diabetes mellitus and decrease lipid oxidation and plasma free fatty acid levels thereby improving the cardiovascular risk profile in type 2 diabetic patients. In the U.K. Prospective Diabetes Study, the treatment with metformin showed a significant reduction in diabetes-related death, heart attacks and stroke (Wright et al., 1998).

The metabolic actions of metformin on cells include increases in insulin sensitivity of responsive tissues, IR number and affinity in skeletal muscle and adipose cells, conversion of glucose to lactate by erythrocytes, tyrosine kinase activity and glucose. Metformin results in a decrease in glucose absorption by gut, plasma glucagon levels, gluconeogenesis and glycogenolysis in the liver.

It was shown that metformin activates the AMPK cascade, which in turn takes part in the glucose transport into the muscle cell (Fryer et al., 2002). Indeed, the exposure to metformin was shown to lead to an increased tyrosine kinase activity and 2-deoxy glucose transport in a dose-dependent manner in rat smooth vascular smooth muscle cells suggesting enhanced sensitivity to insulin and IGF-1 (Dominguez et al., 1996).

Metformin may inhibit hepatic gluconeogenesis in an LKB1-independent and AMPK-independent manner [**21**].

A reduction in gluconeogenesis may occur both ways, in an AMPK-dependent and an AMPK-independent manner. Metformin specifically inhibits complex I of the mitochondrial respiratory chain, suggesting that this inhibition may activate AMPK by increasing the cellular AMP: ATP ratio [**16**,**36**–**38**]. AMPK is a major cellular regulator of lipid and glucose metabolism.

Finally, activated AMPK results in an increase in glucose uptake in skeletal muscle by increasing the GLUT4 (encoded by gene *SLC2A4*) translocation activity [**15**]. The overall pharmacological effect of AMPK activation in the liver includes the stimulation of fatty acid oxidation with inhibition of cholesterol and triglyceride synthesis. Peripheral effects include the stimulation of fatty acid oxidation and glucose uptake in skeletal muscle as well as a systemic increase in insulin sensitivity [**37**]. However, the role of metformin in insulin-mediated glucose uptake was debated [**39**].

A key perturbation in women with PCOs is excessive ovarian androgen production and consequently, excessive estrogen production by GCs. Although the improvement in androgen concentrations in the circulating and follicular environment of women with PCOs treated with metformin may bring indirect beneficial effects to the developing oocytes (Moghetti et al., 2000; Palomba et al., 2010), metformin could change the metabolic capacity of ovarian GCs (Lee et al., 2012).

Polycystic ovary syndrome (PCOs) is a condition of primary ovulatory dysfunction associated with metabolic disturbances. 

It was established that the GCs metabolize glucose in normal human ovaries by glycolysis and this results in the production of lactate and pyruvate (Billing et al., 1983; Harlow et al., 1987; Boland et al., 1993).

Indeed, Purcell et al. have demonstrated blunted insulin-stimulated glucose uptake in vitro by cumulus cells from women with PCOs (Purcell et al., 2012). The previous researchers have also shown that there is an impaired insulin-stimulated lactate production but from intact insulin-responsive steroid production by follicular GCs from women with PCOs (Lin et al., 1997; Fedorcsak et al., 2000; Rice et al., 2005). Previous studies which have cultured GC over 48 h in vitro have demonstrated that insulin-induced glucose uptake by GCs in response to increasing doses of insulin is much lower in GCs from anovulatory patients with PCOs than from women with either normal ovaries or ovulatory polycystic ovaries (Rice et al., 2005).

Tyrosine phosphorylation of the insulin receptor (IR) activates the whole insulin cascade and results in glucose uptake by the cells. Serine phosphorylation of the IR may inhibit the insulin excessive serine phosphorylation which was attributed to the post-binding defect and insulin resistance observed in these women (Prelevic, 1997; Shulman, 2000; Poretsky et al., 2001; Seto-Young et al., 2003).

Metformin alone decreases the fasting plasma glucose concentration to 4–4.5 mM and HbA1c by 1.5–2.0% in patients with type 2 diabetes mellitus.

The metabolic actions of metformin on cells include increases in insulin sensitivity of responsive tissues, IR number and affinity in skeletal muscle and adipose cells, conversion of glucose to lactate by erythrocytes, tyrosine kinase activity and glucose uptake (Cusi and DeFronzo, 1998; Musi et al., 2002). Metformin results in a decrease in glucose absorption by gut, plasma glucagon levels, gluconeogenesis and glycogenolysis in the liver (**Fig. 1**).

It was shown that metformin activates the AMPK cascade, which in turn takes part in the glucose transport into the muscle cell (Fryer et al., 2002) (**Fig. 2**). Indeed, the exposure to metformin was shown to lead to increased tyrosine kinase activity and 2-deoxy glucose transport in a dose-dependent manner in rat smooth vascular smooth muscle cells suggesting an enhanced sensitivity to insulin and IGF-1 (Dominguez et al., 1996).

**Fig. 1:**
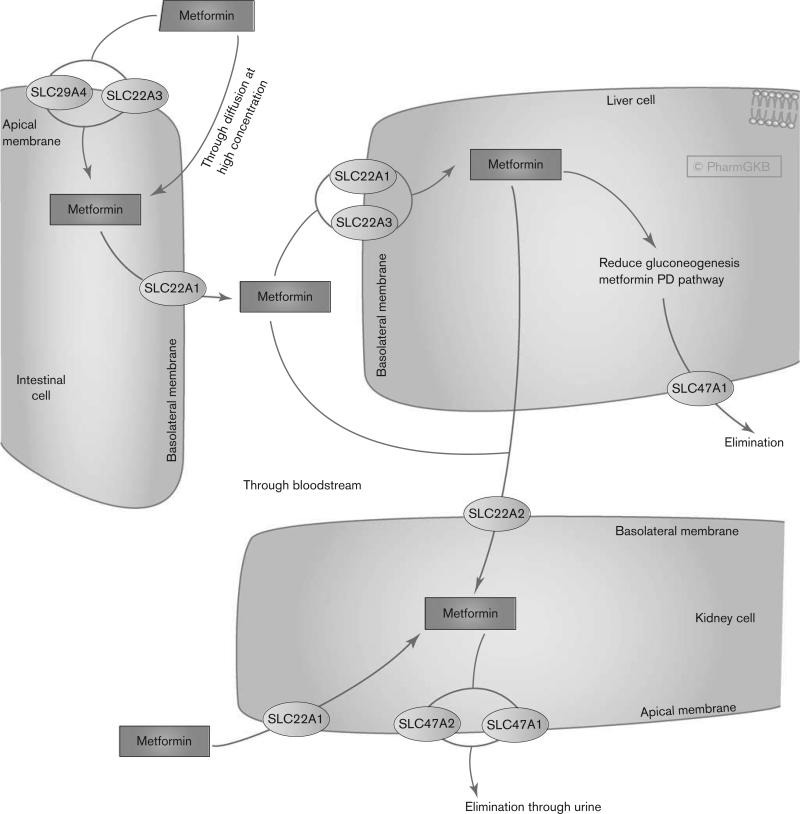
Metformin results in a decrease in glucose absorption by gut, plasma glucagon levels, gluconeogenesis and glycogenolysis in the liver

**Fig. 2:**
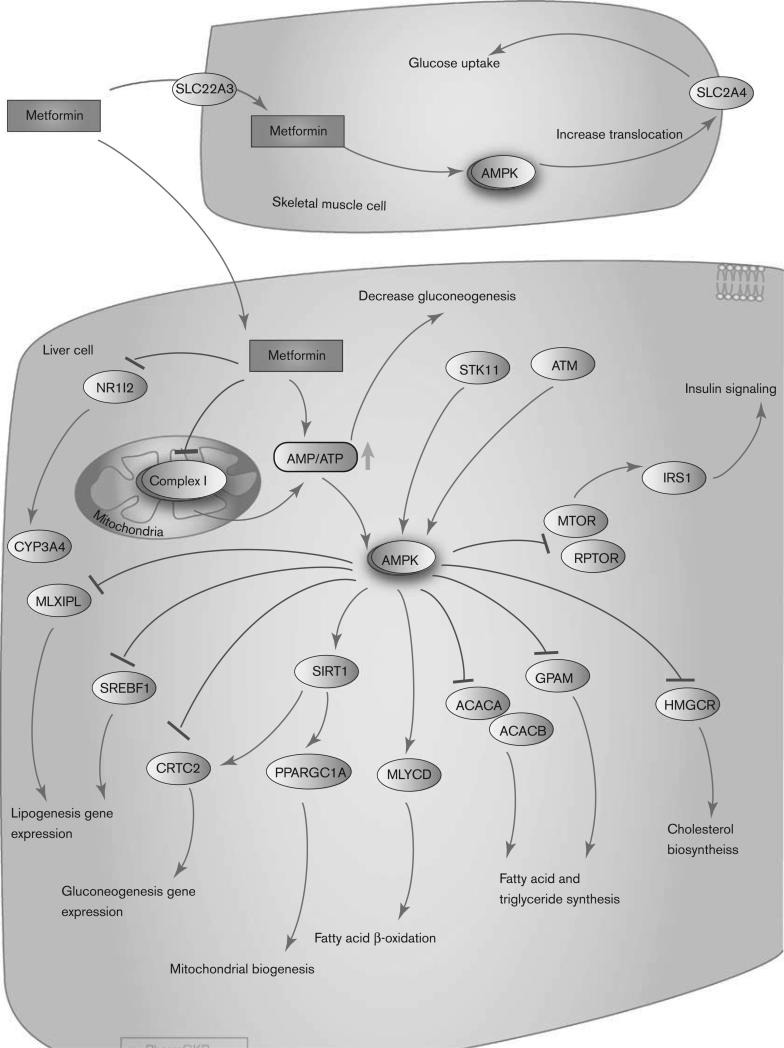
Metformin activates the AMPK cascade, which takes part in the glucose transport into the muscle cell
